# Perceived Barriers to Colorectal Cancer Screening among Eligible Adults in Qatar and the Associated Factors: A Cross-Sectional Study

**DOI:** 10.31557/apjcp.2021.22.1.45

**Published:** 2021-01

**Authors:** Ayman Al-Dahshan, Shaikha Abushaikha, Mohamad Chehab, Mohamed Bala, Vahe Kehyayan, Mieaad Omer, Omayma AlMohamed, Noora Al-Kubaisi, Nagah Selim

**Affiliations:** 1 *Department of Medical Education, Community Medicine Residency Program, Hamad Medical Corporation, Doha, Qatar. *; 2 *Department of Preventive Screening Program, Primary Health Care Corporation, Doha, Qatar. *; 3 *University of Calgary in Qatar, Doha, Qatar. *; 4 *Department of Clinical Affairs, Primary Health Care Corporation, Doha, Qatar. *; 5 *Department of Family and Community Medicine, Primary Health Care Corporation, Doha, Qatar. *

**Keywords:** Barriers, colorectal cancer, screening, Qatar

## Abstract

**Background:**

In Qatar, colorectal cancer (CRC) is the second most common cancer and is projected to be more than triple by 2035. Therefore, CRC periodic screening is vitally important because early detection will improve the success of treatment. In 2016, Qatar established a population-based screening program for CRC targetting average-risk adults. This study aimed to determine the perceived barriers to undergo CRC screening in eligible adults in Qatar and the associated factors.

**Methods:**

This was a cross-sectional study of individuals aged 50-74 years who have been never screened, across six primary health centers between September 2018 and January 2019. A non-probability sampling method was used to recruit participants. Participants were interviewed using a structured questionnaire. Descriptive and analytic statistics were applied.

**Results:**

A total of 188 individuals participated in the study. The mean age of the participants was 58.3 (SD ±6.4) years. Most participants were females (54.5%) and non-Qatari Arabs (54.3%). The top five reported barriers to CRC screening were: not at risk due to absence of symptoms (60.6%), not at risk due to absence of family history (55.1%), not at risk due to adopting a healthy lifestyle (52.7%), lack of time (41%), and lack of reminders by healthcare workers (39.4%). Bivariate analyses identified statistically significant associations between certain barriers and female gender, nationality, and educational level (primary school and below).

**Conclusion:**

The present study identified several barriers to undergoing CRC screening among eligible adults in Qatar. Such results provide a basis for tailoring of future educational campaigns that are relevant, specific, and appealing to such a cohort.

## Introduction

Colorectal cancer (CRC) is the third most common cancer globally. It accounts for 1.8 million cases annually. It is also the second-leading cause of cancer mortality with 862,000 deaths each year (World Health Organization, 2018). Regionally, the incidence of CRC varies more than ten folds with the highest incidence being recorded in Australia and New Zealand while the lowest being reported in South-Central Asia and Africa (Global Burden of Disease Cancer Collaboration, 2017). In the Eastern Mediterranean Region, it is estimated that CRC has the highest incidence rate (mean= 10.19 ± 5.30) of all cancers (Sabzalizadeh-Ardabili et al., 2019). In Qatar, CRC was the second most common cancer (10.23% of all cancer cases) and ranked third for cancer-related deaths (12.66%) during 2015 (Ministry of Public Health, 2017b). Also, more than two-thirds of CRC cases were diagnosed at an advanced stage. As a result, the National Cancer Framework 2017-2022 aims at increasing the proportion of CRC patients diagnosed in stages 1 and 2 while achieveing a 70% uptake of the national CRC screening program (p. 27). 

Like other types of cancer, CRC has a multifactorial etiology. The most notable risk factors for developing CRC include the male gender, certain races, obesity, age (≥50 years), a family history of hereditary CRC, medical history of adenomatous polyps, inflammatory bowel disease, diabetes, certain dietary habits (e.g., consumption of red or processed meat), and social history (e.g. tobacco use, consumption of alcohol) (Macrae, 2020). Since most cases of CRC originate from adenomatous polyps that slowly progress to malignant lesions, periodic screening has been recommended to identify the disease at an early stage and decrease the associated mortality (Yang et al., 2014). The identification of precancerous polyps can also help prevent the occurrence of the disease. 

Despite the numerous benefits of screening, several barriers to undergo regular screening for CRC continue to exist. A study among 10,078 screening eligible participants in Hong Kong showed that financial hardship, screening-induced discomfort or bodily harm, limited access to the screening services, embarrassment or anxiety due to screening, and time limitations were the top barriers against CRC screening (Wong et al., 2013). Another study conducted among Korean individuals aged 50 years or over identified the following barriers to CRC screening: absence of symptoms, inconvenience of procedure, decreased level of knowledge, lack of trust in screening test, and fear of test result (Lee, 2018). Moreover, a study conducted among Pakistani medical residents found that the absence of screening facilities was one of the major barriers to CRC screening (Hasan et al., 2017). In most Arab countries there is a lack of population-based screening programs as a result of cultural and religious barriers as well as low levels of education (Arafa and Farhat, 2015). A study among primary care physicians and nurses in Oman showed that the lack of awareness was the main patient-related barrier to undergoing CRC screening. On the other hand, the most commonly cited system-based barriers were the lack of screening-related policies or clinical protocols, deficient training of health care professionals, and delayed screening appointments (Muliira et al., 2016). 

In the State of Qatar, the Primary Health Care Corporation (PHCC), the country’s main provider of primary health services, has introduced two population-based screening programs for colorectal and breast cancers themed “Screen for Life” since 2016. Regarding CRC, the program targets average-risk adults aged between 50 and 74 years through fecal immunochemical testing (FIT) (Primary Health Care Corporation, 2018). Average-risk adults are those who are “without conditions such as inflammatory bowel disease, familial adenomatous polyposis (FAP), hereditary nonpolyposis colorectal cancer (HNPCC), or positive family history of colorectal neoplasia (adenoma or CRC)” (Anderson et al., 2002). Eligible candidates access the screening service by invitation from the dedicated cancer screening call center, physician referral, or self-referral. If the result of the screening is positive, the patient is referred to undergo colonoscopy at a secondary care hospital of the Hamad Medical Corporation (Primary Health Care Corporation, 2016). Given that the incidence of CRC in Qatar is projected to be more than triple by 2035 (Ministry of Public Health, 2017a), it is associated high mortality rates, and early detection is recognized to increase the chance of successful treatment, it is of utmost importance to determine the barriers to screening uptake. Therefore, the purpose of this study was to determine the perceived barriers to undergo CRC screening in eligible adults in Qatar and the associated factors.

## Materials and Methods

*Study design and setting*

This was a cross-sectional study. It was conducted in six primary health care (PHC) centers in Qatar. These centers are the population’s first line of contact with Qatar’s health care system. Each center serves a large population of various educational, ethnic, cultural, and social backgrounds representing the overall community in Qatar. Two PHCs were selected from each of the North, West and Central geographic regions in the country. The data collection process carried out between September 2018 and January 2019.

*Study population and sampling*

The study population included all patients of the selected PHC centers between the ages of 50 and 74 years, English or Arabic speaker, any gender or nationality, and who have never been screened for CRC. The researchers excluded those who were incapable of communicating or giving consent due to any disability. Potential participants were recruited through a convenient sampling method. Each potential candidate for the research was asked relevant questions to establish their eligibility for the purpose of the study.

*Sample size*

Based on Qatar’s 2015 Census, about 211,207 individuals aged 50 years and over inhabited the country during that year. Thus, the calculated sample size was 196 individuals with a 95% confidence interval, a margin of error of 7%, and a hypothesis that 50% of the sample reported having a barrier to undergoing screening for CRC. 

*Data collection*

The data collection process relied on an interview-based questionnaire as described below. Subjects were recruited and enrolled consecutively by trained resident physicians who were located in the main waiting areas of the health centers. With the support of health center staff they identified eligible adults. Potential participants were explained about the purpose and nature of the study and were invited to participate. Written consents were obtained from all participants. Participants were interviewed in their preferred language of Arabic or English for 10 to 15 minutes. After concluding the interview, each participant received an educational pamphlet about CRC screening, the process of undergoing screening, and the benefits of regular CRC screening. Any participant who presented a potential symptom or concern regarding CRC was advised to inform his/her primary care physician for further assessment and follow up. 

*Data collection tool*

The current study employed a structured questionnaire (see in Appendix). It achieved face and content validity by extensive search of the literature, and critical review by an expert panel made up of community medicine consultants and bowel cancer experts in Qatar. The questionnaire was translated and back-translated (English-Arabic) by two independent professional translators and any differences were resolved by consensus. In addition, a pilot study was conducted with 20 participants and modifications were made as necessary. 

The questionnaire consisted of two main sections. Section A consisted of 6 questions exploring the background characteristics of the participants (age, gender, nationality, marital status, level of education, employment status). Section B included 11 closed-ended questions about perceived barriers to CRC screening. In interviewing the patients about barriers to CRC screening, they were asked “to what extent do you think the following factors could form barriers to undertake annual CRC screening?” Their responses were based on a three-point Likert scale: Agree, Neutral, and Disagree.

*Statistical analysis*

The Statistical Package for the Social Sciences-SPSS version 23 was used for statistical analysis. Both descriptive and analytic statistics were applied as appropriate. The level of statistical significance was set at 0.05. Frequencies and percentages were calculated for categorical variables while means and standard deviations were calculated for continuous variables. The Chi-Square test of independence was used to determine if there was any significant relationship between explanatory and the outcome variables. 

## Results

*Background characteristics*

A total of 225 eligible candidates were invited to participate in the current study. Among these, 197 participants agreed to participate (response rate of 87.5%). Nine questionnaires were discarded due to incompleteness with 188 questionnaires remaining for the final analysis. The mean age of the participants was 58.3 (SD ±6.4) years old. Most respondents were females (54.5%) and non-Qatari Arabs (54.3%). Table 1 presents the background characteristics of the study participants. 

*Barriers*

**Figure 1.**
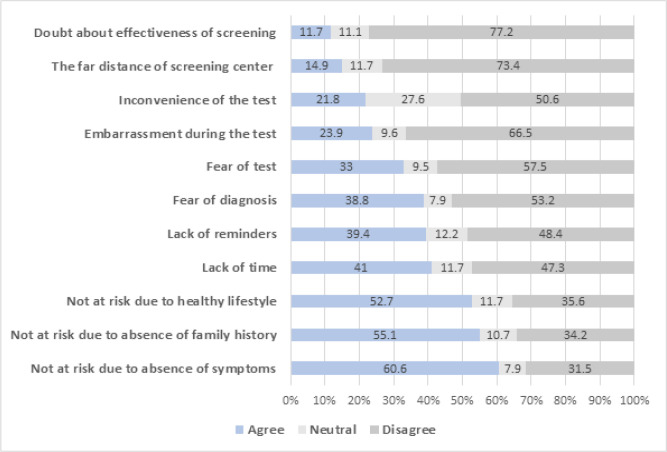
Frequency of Perceived Barriers to Colorectal Cancer Screening among Study Participants (N=188)

Regarding the different barriers examined in this study, the most common ones reported by the participants were not perceived at risk due to the absence of symptoms related to CRC (60.6%), not perceived at risk due to the absence of a family history of CRC (55.1%), not perceived at risk due to their healthy lifestyle (52.7%), the lack of time (41%), and the lack of reminders by healthcare workers (39.4%). On the other hand, the least frequently reported barrier was the participants’ doubt about the effectiveness of the colorectal cancer screening test (11.7%). Figure 1 shows the distribution of reported barriers.

*Barriers by gender*

In bivariate analysis, using the Chi-Square test of independence, a statistically significant relationship was found between certain barriers examined and gender. For instance, female participants were significantly more likely to identify the fear of diagnosis (47.1% vs. 29.4%, p=0.01), fear of the screening test (43.1% vs. 20.0%, p=0.01), embarrassment during the test (29.4% vs.16.5%, p=0.02), and the far distance of the screening facility (20.6% vs. 8.2%, p=0.01) as barriers to CRC screening compared to their male counterparts (Table 2). 

*Barriers by educational levels*

As shown in Table 3, those with higher educational levels (secondary school and above) were less likely to report barriers than their peers. A statistical significance was found between the educational level and all the surveyed barriers except for the lack of time, lack of reminders, inconvenience of the test, and the remoteness of the screening facility. 

*Barriers by Nationality*

Regarding the nationality variables and the type of barrier, as shown in Table 4, Qataris were more likely to report the fear of diagnosis (50%), embarrassment during the screening test (35.2%), inconvenience of the test (33.3%), and doubt about its effectiveness (16.7%) than their expatriate peers. In addition, among the expatriate population, Arabs were more prone to report embarrassment during the screening test (25% vs. 17.6%, p<0.05) and inconvenience of the test (21.9% vs. 15.7%, p<0.05) as barriers than their non-Arab counterparts. 

**Table 1:** Background Characteristics of Participants Reported Barriers to Screening for Colorectal Cancer (N=188)

Variable	N (%)
Age (mean ± SD)	58.3 ±6.4
Gender	
Male	85 (45.5)
Female	102 (54.5)
Nationality	
Qatari	54 (28.7)
Non-Qatari Arab	102 (54.3)
Non-Arab	32 (17.0)
Marital status	
Married	166 (88.3)
Unmarried	22 (11.7)
Level of education	
Primary school and below	52 (27.8)
Secondary school and above	135 (72.2)
Employment status	
Employed	84 (44.7)
Retired	104 (55.3)

**Table 2:** Gender Differences Regarding the Reported Perceived Barriers to Undergoing Colorectal Cancer Screening (N=188)

Type of the perceived barrier	Gender		p-value
	Male (%)	Female (%)	
Not at risk due to absence of symptoms	67.1	54.9	0.06
Not at risk due to healthy lifestyle	52.9	52	0.5
Not at risk due to absence of family history	50.6	58.4	0.17
Lack of time	36.5	44.1	0.18
Lack of reminders	32.9	44.1	0.07
Fear of diagnosis	29.4	47.1	0.01*
Fear of test	20	43.1	0.01*
Embarrassment during test	16.5	29.4	0.02*
Inconvenience of test	21.2	22.5	0.48
Doubt about effectiveness of screening	9.4	13.7	0.12
The far distance of screening center	8.2	20.6	0.01*

*Statistically significant (p-value ≤ 0.05)

**Table 3:** Relationship between Educational Level and the Reported Perceived Barriers to Undergoing Bowel Cancer Screening (N=188)

Type of the perceived barrier	Educational level		p-value
	Primary school and below (%)	Secondary school and above (%)	
Not at risk due to absence of symptoms	73.1	56.3	0.02*
Not at risk due to healthy lifestyle	71.2	45.9	0.01*
Not at risk due to absence of family history	66.7	51.1	0.04*
Lack of time	42.3	40.7	0.48
Lack of reminders	38.5	40.0	0.49
Fear of diagnosis	50.0	34.8	0.04*
Fear of test	44.2	28.9	0.03*
Embarrassment during test	38.5	18.5	0.01*
Inconvenience of test	30.8	18.5	0.05
Doubt about effectiveness of screening	19.2	8.9	0.04*
The far distance of screening center	17.3	14.1	0.36

* Statistically significant (p-value ≤ 0.05)

**Table 4:** Nationality Differences Rregarding the Reported Perceived Barriers to Undergoing Colorectal Cancer Screening (N=188)

Type of the perceived barrier	Nationality			p-value
	Qatari (%)	Non-Q Arab (%)	Non-Arab (%)	
Not at risk due to absence of symptoms	63.0	57.8	65.6	0.67
Not at risk due to healthy lifestyle	53.7	51.0	56.3	0.85
Not at risk due to absence of family history	61.1	51.0	58.1	0.45
Lack of time	42.6	39.2	43.8	0.86
Lack of reminders	37.0	44.1	28.1	0.24
Fear of diagnosis	50.0	38.2	21.9	0.03*
Fear of test	44.4	28.4	28.1	0.1
Embarrasment during test	35.2	17.6	25.0	0.04*
Inconvenience of test	33.3	15.7	21.9	0.04*
Doubt about effectiveness of screening	16.7	9.8	9.4	0.05*
The far distance of screening center	20.4	13.7	9.4	0.34

* Statistically significant (p-value ≤ 0.05)

## Discussion

To the best of our knowledge, this was the first study in the State of Qatar to determine the barriers to undergoing CRC screening in asymptomatic average-risk adults who were eligible for CRC screening . Our study found that the main barriers for undergoing CRC screening varied in frequency with the most reported being the absence of symptoms, absence of family history, leading a healthy lifestyle, time constraint and lack of reminders or recommendation from their healthcare providers. Also, a statistically significant association was found between certain barriers and female gender, nationality, and educational level (primary school and below).

Regarding the reported barriers, our study findings are comparable with previously published literature. For instance, a recent systematic review that included a total of 23 studies conducted in Asia concluded that low level of education, negative perceptions towards screening, fear of the test results, financial issues, lack of time, lack of physicians’ recommendation, difficulty in accessing screening facilities, fear of the diagnosis, and low perceived risks were major barriers to CRC screening (Azeem et al., 2016). Furthermore, a study conducted in Saudi Arabia by Al-Hajeili et al., (2019) concluded that fear of the procedure, absence of symptoms, and fear of the results were the most commonly reported barriers for seeking CRC screening. Another study conducted in Pakistan among screening-eligible adults found that more than 40% of the participants reported being afraid of abnormal test results, 29% reported lack of time, and about 27% reported feeling embarrassed being screened which is comparable to our finding of 24%. 

Another major reported barrier to screening was the lack of reminders from healthcare providers This finding is consistent with the findings of a study from Oman that about two-thirds of primary HCPs rarely recommended CRC screening to eligible adults (Muliira et al., 2016). To raise awareness of CRC screening, a multi-center study of the Asian-Pacific region highlighted the important role of physicians as a predictor of screening uptake among eligible patients (Koo et al., 2012). Similarly, a survey of medical students in Saudi Arabia showed a limited knowledge of colorectal cancer risk factors and a poor attitude towards screening (Althobaiti and Jradi, 2019). The researchers suggest targeted education of medical students, clinicians, and general population of CRC risk factors and the importance of CRC screening. 

In contrast to our results, a cross-sectional study among primary care patients in the United States revealed that the most commonly reported barriers to CRC screening were the fear of the procedure, lack of transportation, and financial difficulty (Muthukrishnan et al., 2019). However, as the national CRC screening in Qatar is provided free of charge to all residents and nationals , the financial constraint is not perceived as a possible barrier. 

The aforementioned results can be explained using the Health Belief Model (HBM) which was developed to describe and predict health-related behaviors such as the uptake of health services (Jones et al., 2015). According to the model, four factors influence individuals’ behaviors about preventing an illness: “perceived susceptibility” (referring to the individual’s perceived risk of acquiring a disease). “perceived severity” of the negative consequences of the condition, “perceived benefits” of taking corrective action related to the condition, and finally, “perceived barriers” that prevent them from taking the necessary corrective action. Our data concern two of these factors, the perceived susceptibility of getting CRC and the perceived barriers to accessing CRC screening. The majority of the participants felt that they were not susceptible of acquiring CRC due to not having symptoms (60.6%), not having a family history (55.1%), and having a healthy lifestyle (52.7%). 

Moreover, our study analyzed the relationship between selected sociodemographic factors and the perceived barriers to undergoing CRC screening among the participants. As a result, the female gender, nationality and educational level (primary school and below) variables were significantly associated with certain barriers such as fear of the screening test and related embarrassment. The aforementioned findings correlate with the earlier established gender gap in which men were more likely to undergo CRC screening than women. This necessitates the development of gender-specific strategies to increase the awareness of CRC screening among women (Yager S, 2011). 

In addition, there was a statistically significant difference in the distribution of reported barriers by nationality in the current study. However, the percentage difference between the nationals and expatriates was not clinically significant given that the healthcare system is equally accessible to both cohorts (Hussin A, 2010). Finally, the significant association between a low education level (below secondary school) and decreased uptake of CRC screening corroborates with earlier research (Azeem et al., 2016). 

The educational level is a non-modifiable risk factor and is associated with the participant’s health literacy level. Thus, future awareness interventions should focus on delivering educational materials in lay language and through multiple channels (newspapers, videos, pamphlets, radio, leaflets, brochures). Also, individual as well as group education sessions will help accommodate the different educational backgrounds of eligible participants in the country (Gimeno Garcia, 2012). It would also be necessary to educate healthcare professionals about the importance of screening and the barriers that eligible adults perceive preventing them from accessing life saving screening services. 

This study, however, should be interpreted cautiously due to a few limitations. One limitation is that the recruitment of the participants was based on a convenient sampling technique in the PHC centers. This could affect the generalizability of the results to the local community in Qatar. Additionally, the cross-sectional design of the current study limits its interpretation of the causal relationship between the variables. Despite the limitations, the present results provide a baseline for further amplification of the national CRC screening program in the State of Qatar.

In conclusion, the present study has identified several perceived barriers to undergoing CRC screening in eligible adults in Qatar. Such results provide a basis for the tailoring of future educational campaigns that are relevant, specific, and appealing to such a cohort. Such efforts would help in achieving the national target of 70% uptake of screening services. 

*Author contribution statement*

AAD: Conceptualization, Methodology, Formal analysis, Investigation, Writing - Original Draft SA: Supervision. MC: Investigation, Writing - Original Draft MB: Investigation, MO: Investigation, OA: Investigation NAK: Methodology NS: Methodology, Project administration VK: Writing- Reviewing and Editing.

## Data accessibility

Data are available upon reasonable request.
